# Flame Retardancy and Mechanism of Novel Phosphorus-Silicon Flame Retardant Based on Polysilsesquioxane

**DOI:** 10.3390/polym11081304

**Published:** 2019-08-04

**Authors:** Shengjie Zhu, Weiguang Gong, Ji Luo, Xin Meng, Zhong Xin, Jie Wu, Zewen Jiang

**Affiliations:** Shanghai Key Laboratory of Multiphase Materials Chemical Engineering and Production Engineering Department, School of Chemical Engineering, East China University of Science and Technology, Shanghai 200237, China

**Keywords:** PLA, flame retardancy, phosphorus-silicon synergism, mechanism

## Abstract

A novel phosphorus-silicon flame retardant (P_5_PSQ) was prepared by bonding phosphate to silicon-based polysilsesquioxane (PSQ) and used as flame retardant of poly (lactic acid) (PLA). The results show that PLA with 10 wt % P_5_PSQ has a limiting oxygen index (LOI) 24.1%, the peak heat release rate (PHRR) and total heat release (THR) of PLA decrease 21.8% and 25.2% compared to neat PLA in cone calorimetric test, indicating that P_5_PSQ shows better flame retardancy in comparison to PSQ. Furthermore, the study for the morphology and composition of carbon residue after the combustion of PLA and the gas release of PLA during combustion illustrate that P_5_PSQ has flame retardancy in condensed phase and gas phase simultaneously. In condensed phase, phosphorus from phosphate promotes the formation of more stable and better carbon layer containing Si and P, which inhibits the transfer of heat and oxygen in the combustion. In gas phase, the phosphate in P_5_PSQ emits phosphorus-containing compound that can restrain the release of C–O containing products, which may have effective flame retardancy for PLA in gas phase to a certain extent. In one word, P_5_PSQ is denoted as a good phosphorus-silicon synergistic flame-retardant.

## 1. Introduction

With the shortage of oil and the problem of “white pollution”, biodegradable plastic poly(lactic acid) (PLA) has become a well-received alternative to petroleum-based plastics due to its equivalent mechanical properties and process ability, and its production cost is decreasing continuously owing to the continuous advancement of synthetic technology [1,2]. The application of PLA is not limited to the fields of issue engineering, drug release and other biomedical fields, and it is extending to the fields of textiles, tableware, and packaging. PLA is becoming the most promising biodegradable plastic. However, PLA is highly flammable, which limits its application in many other fields on account of a thin visible carbonized layer produced during the combustion [3]. Therefore, research on the flame-retardant modification of PLA is of great significance in broadening its potential application in electronic materials, automobiles, and aircrafts [4,5].

Halogen-based flame-retardants show excellent flame retardant property, but they produce toxic gases that are harmful to people’s health during the combustion. In modern society, people are paying more and more attention to the environmental properties of additives, so halogen-based flame-retardants are restricted obviously and the research on high-efficient halogen-free flame-retardant systems has become a fundamental issue to increase the flame retardant property of PLA [6,7]. Although the inorganic flame retardant has good stability and low toxicity of the flue gas, the large amount addition will deteriorate the processing and mechanical properties of polymers due to the obvious difference between flame retardant and the matrix. As a result, this kind of flame retardant is not used to all kinds of polymers extensively. Recently, intumescent flame retardant (IFR) as novel halogen-free flame-retardant has the characteristics of low smoke, low toxicity, and no corrosive gas generation and has becoming a good candidate for PLA, but it still has some problems that limit the application. First, the addition amount is also high, and it affects the processing of the PLA. Then the important component of acid source easily absorbs water [8], and it affects the stability of PLA [9,10,11]. Under these conditions, it is very important to design a novel halogen-free flame-retardant system for PLA.

The silicon-based flame retardant is becoming a novel typical representative of halogen-free flame retardant, because the presentation of silicon in polymer is beneficial to the formation of stable heat-insulating carbon protective layer including –Si–O– and –Si–C– bonds, which is very important for the improvement of flame retardancy of polymer [12]. Some studies showed that functionalized polysilsesquioxanes is a representative of silicon-based flame retardant, which can be used as flame retardant to improve the flame retardancy of polyesters such as polycarbonate [13,14,15]. PLA is a kind of polycarbonate, so the polysilsesquioxanes can be used to increase the flame retardancy of PLA too. Furthermore, the research in our group shows that the groups in polysilsesquioxanes has different aspects for the flame retardant properties of polysilsesquioxanes through the influence for the migration of flame retardant, which makes the polysilsesquioxanes with different groups show different flame retardancy [16]. As a result, in order to improve the flame retardancy of polysilsesquioxanes, it is very important to increase the stability and amount of carbon layer through improving the migration of silicon. It is reported that the silicon-based flame retardant embodies better flame retardant property when it is used with phosphorus-based flame retardant, which is related to two reasons. In one side, the introduction of phosphorus is beneficial to the information of impact and protective char layer, which efficiently protects the inner material from being attacked by the heat and flame in combustion [17,18]. In another side, phosphorus is beneficial for the migration of silicon to the surface of carbon layer, then form more stable carbon protective layer [19,20,21]. Based on the research mentioned above, it is reasonable and imperative to design a phosphorus-silicon synergistic flame retardant system through the introduction of phosphorus to polysilsesquioxanes system. 

In addition, Wei et al. [22] found that the synergistic effect of the phosphorus-silicon flame-retardant system could be further improved when the two flame-retardant elements of phosphorus and silicon were designed in the same molecule [23,24]. Therefore, in this study, based on the preparation of poly(amino-epoxy)silsesquioxane (PSQ), the 2,2’-methylenebis(4,6-di-tert-butyl-phenyl)phosphate group was bonded into PSQ to build a phosphorus-silicon synergistic flame retardant system (P_5_PSQ). Then the effects of phosphate on the flame retardancy and mechanism of PSQ in PLA were studied in detail to regulate the design and application of phosphorus-silicon synergistic flame retardant system.

## 2. Experimental Section

### 2.1. Materials

Phosphorus oxychloride (POCl_3_) and anhydrous ethanol (C_2_H_5_OH) were provided by Sinopharm Chemical Reagent Co., Ltd. (Shanghai, China). 2,2’-methylenebis(4,6-di-tert-butylphenol) was purchased from Nantong Advanced Chemical Co., Ltd.(Nantong, China). Toluene(TL) and Triethylamine (TEA) were supplied by Shanghai Lingfeng Chemical Reagent Co., (Shanghai, China). 3-(2,3-Epoxypro-propyl)trimethoxysilane (EPTMS) and (3-Aminopropyl) trimethoxysilane (APTMS) were produced by Nanjing Capatue Chemical Co. (Nanjing, China) and Hubei Debang Chemical New Material Co. Ltd. (Wuhan, China),respectively. PLA (2003D) was purchased from NatureWorks LLC (Blair, NE, USA).

### 2.2. Synthesis of the Phosphorus-Silicon Flame Retardant (P_5_PSQ)

**Scheme 1:**
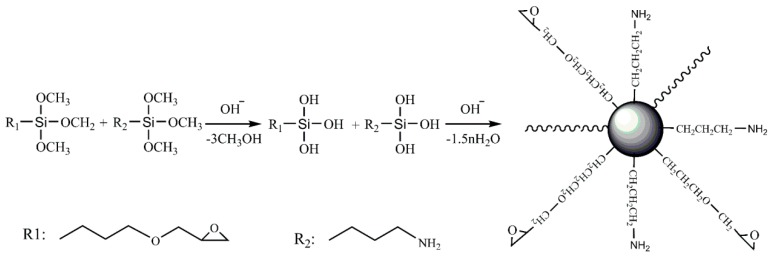
Synthesis route of PSQ.

Preparation of poly(amino-epoxy)silsesquioxane (PSQ): 6 mL of EPTMS was dissolved into 150 mL deionized water in magnetic stirring reactor, and then 4 mL of APTMS was added dropwise in 3 min. The reaction was carried at room temperature for 8 h. Then the product was filtered. Finally, the product was washed three times with deionized water and absolute ethanol, respectively. The resulting white filter product was freezing-dried for 12 h to obtain PSQ. Product yield is about 82%. The synthesis route is shown in Scheme 1.

**Scheme 2:**
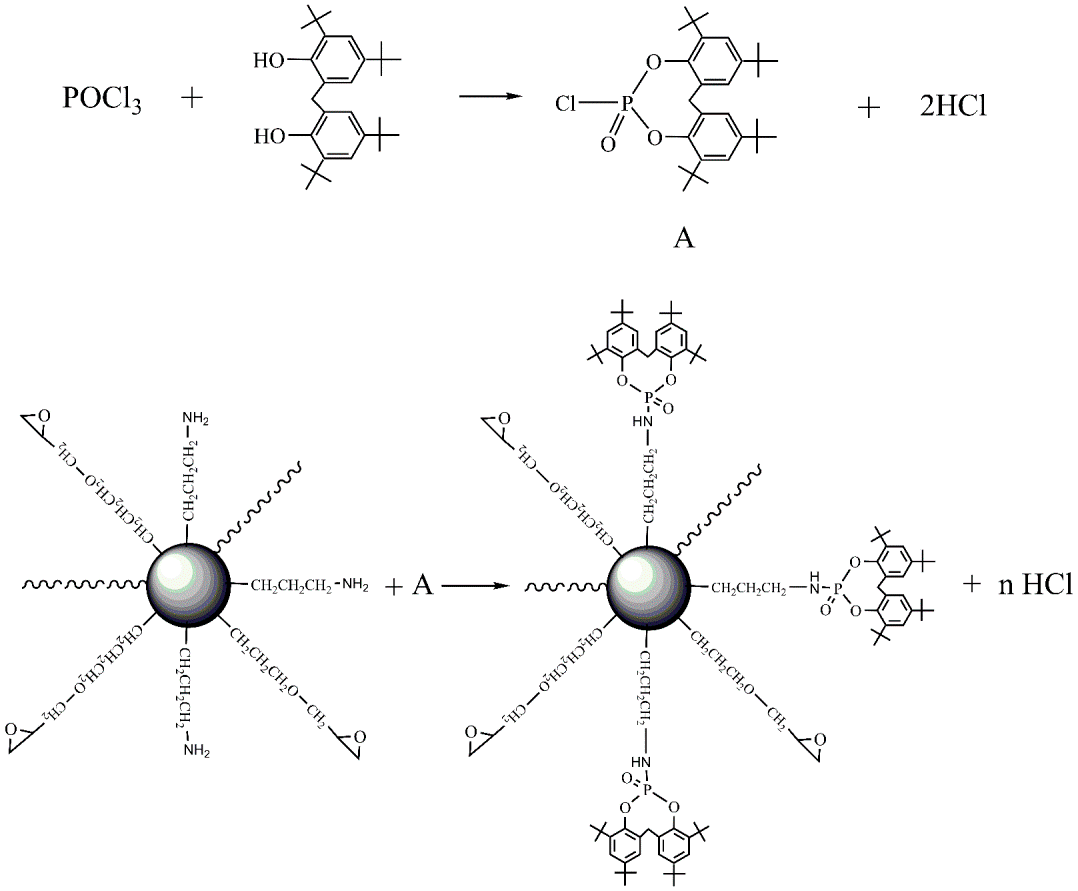
Synthesis route of P_5_PSQ.

Preparation of phosphate-containing polysilsesquioxane (P_5_PSQ): In a 250 mL glass flask under nitrogen atmosphere, equipped with a magnetic stirrer, a thermometer, a spherical condenser and heating bath, 10.7 g (0.025 mol) 2,2′-methylenebis(4,6-di-tert-butylphenol), 50 mL toluene, and 10.4 mL (0.075 mol) triethylamine (TEA) were added successively. After the mixture was dissolved and mixed fully, 20 mL of a toluene solution containing 3 mL (0.0328 mol) phosphorus oxychloride (POCl_3_) was introduced dropwise at a rate of approximately 2 to 4 drops per second. After the reaction was carried at 15 to 20 °C for 3 h, 15 g PSQ was added to the reaction system. Then the oil bath was heated to 110 °C and maintained at the temperature for 6 h. The final product was filtered and washed three times with toluene and water, respectively. At last, it was dried in a vacuum oven for 12 h to get P_5_PSQ. The calculated grafting rate is about 32%. The synthesis route is as shown in Scheme 2.

### 2.3. Preparation of the Flame-Retardant PLA Composites

The flame-retardant PLA composites were prepared by mixing PSQ and P_5_PSQ into PLA at the adding amount of 10 weight percent. Before processing, PLA pellets were dried in a vacuum oven at 80 °C for 12 h. Then the PLA and additives were blended in a Haake-Buchler batch mixer at 180 °C with a rotor speed of 60 rpm for 10 min. At last, the flame-retardant PLA composites were obtained. 

Preparation of cone calorimetry sample: The processed PLA was packed in a 100 mm × 100 mm × 40 mm mold closely, and the mold was performed using a plate vulcanizer with a pressure of 8–10 MPa at 180 °C. In order to avoid air bubbles, the PLA samples were pressed several times continuously in the hot-pressed layer before starting hot pressing to eliminate possible bubbles in the mold. After hot pressing for 5 min, the mold was transferred to a cold-pressed layer and cooled by the circulating water for 5 min to obtain a proper-shaped sample for cone calorimetric test. In the end, the samples were placed in a desiccator for use.

### 2.4. Measurement and Characterization

The fourier transform infrared spectra (FT-IR) were recorded on a FT-IR spectrometer (Nicolet iS10, Thermo Fisher, Waltham, MD, USA) over the wavenumber range of 400–4000 cm^−1^ using a thin KBr Pill.

^29^SiNMR measurements were performed by 400 MHz WB spectrometer (AVIII, Bruker, Karlsruhe, Germany), and the ^29^Si frequency was 79.49 MHz. 

The thermogravimetric analysis (TGA) were carried out using thermogravimetric analyzer (Q500, TA Instruments, New Castle, DE, USA) at the heating rate of 20 °C/min under nitrogen from room temperature to 800 °C.

Cone calorimetric tests were carried on a FTT Cone Calorimeter (Fire Testing Technology Limited, West Sussex, UK) with the dimension of the sample sheets of 100 mm × 100 mm × 4 mm at a heat flux of 35 kW/m^2^ according to ASTMD-6113.

Limiting oxygen index (LOI) values were determined using an Oxygen Index Instrument (Motis Fire Technology Company, Kunshang, China) according to ASTM D2863, with the dimension of the sample sheets of 130 mm × 6.5 mm × 3 mm.

Elemental analysis was performed using an elemental analyzer (Vario ELIII, Elementar, frankfurt, Germany) to test the contents of nitrogen element.

Inductively coupled plasma-atomic emission spectroscopy (ICPOES) analysis was performed using a spectrometer (ICPS-725, Agilent, Palo Alto, CA, USA) to test the contents of silicon and phosphorus elements.

Thermogravimetric analysis/Fourier transform infrared spectroscopy (TG-IR) measurements were performed on a thermogravimetric analyzer (TGA8000*, PerkinElmer, Waltham, MA, USA) that was linked to the FT-IR instrument (8400S, Shimadzu, Kyoto, Japan), by means of a heated line. The test procedure for TGA was 20 °C/min from room temperature to 600 °C under N_2_ atmosphere. 

The char residues of the flame-retarded PLA composites were obtained from the burned samples. The char residues were analyzed by scanning electron microscopy (SEM) (Nova Nano 450, FE, Hillsboro, OR, USA) coupled with Noran SystemSix energy dispersive spectrometer (EDS) (Noran SystemSix, Thermo Fisher, Waltham, MD, USA) and raman spectrometer (LabRAM HR, HORIBA Jobin, Paris, France) at room temperature, respectively.

The rheological properties of PLA samples were conducted using a rotational rheometer (Discovery HR-3, TA Instruments, New Castle, DE, USA) equipped with parallel plates (2 mm gap and 25 mm diameter). A dynamic frequency sweep (0.05–100 rad/s) was conducted at 180 °C (within the linear viscoelastic region).

## 3. Results and Discussion

### 3.1. The Characterization of P_5_PSQ

The preparation routes of PSQ and P_5_PSQ are shown in Scheme 1 and Scheme 2. The elemental contents of N, P and Si in PSQ and P_5_PSQ were measured by elemental analysis and ICP analysis, and the results are shown in Table 1.

From the results of the elemental compositions, it can be seen that the contents of N and Si of P_5_PSQ show a little decrease compared to those of PSQ. That relates to the introduction of phosphate into the PSQ molecule, because the presentation of phosphate decreases the element ratio of N and Si in the molecule. The results indicate that the group of phosphate bonded to PSQ preliminarily, and further characterizations are as follows.

**Figure 1:**
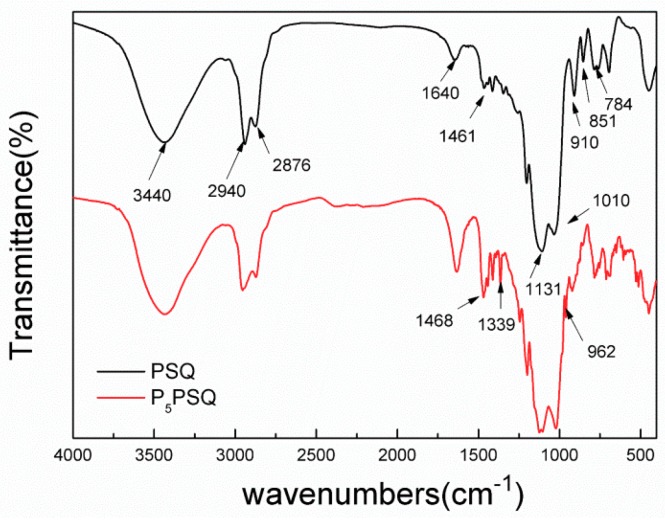
FT-IR spectra of PSQ and P_5_PSQ.

Figure 1 shows the FT-IR spectra of PSQ and P_5_PSQ. It is observed that they have some peaks in common. First, they both have a broad peak centered at 3440 cm^−1^, which is attributed to the absorptions of bending vibration of –Si–OH– and the stretching vibration of –NH_2_ [25]. Then, the peaks at 2940 and 2876 cm^−1^ related to the vibration of C–H in CH_2_ group are showed in the two IR spectra [24]. They both have absorptions bands at 1010, 1131 and 784 cm^−1^, which are assigned to the Si–O–Si stretching vibration. The peak around 1010 cm^−1^ indicates that there is a cage structure in the PSQ, the peak at 1131 cm^−1^ indicates that there is a ladder or layered structure in the PSQ, and the peak at 784 cm^−1^ is ascribed to Si–C bond stretching vibration [25,26,27]. At last, the peaks at 910 and 1461 cm^−1^ are attributed to epoxy and N–H bonds [27,28], and the absorption band at 1640 cm^−1^ corresponds to bending vibration of the hydroxyl group of absorbed water. All the results mentioned above show that P_5_PSQ has the structure of the original PSQ, and the basic structure of the PSQ has not been destroyed during the grafting process of phosphate. 

In addition, some additional absorption peaks were observed in the spectrum of P_5_PSQ, the absorptions band centered at 1468 cm^−1^ with higher intensity indicates the presence of phenyl. The rocking vibration absorption of primary amine at 851 cm^−1^ disappeared, illustrating that the amino group reacted with phosphate. And the peaks at 962 cm^−1^ is assigned to the absorption of P–N–C bond [29]. Furthermore, according to reference [30], we know that the absorption peak of P=O is in the range of 1350–1250 cm^−1^, so the absorption peak at 1339 cm^−1^ is considered to relate to P=O. All these results prove that phosphate was successfully reacted with PSQ.

**Figure 2:**
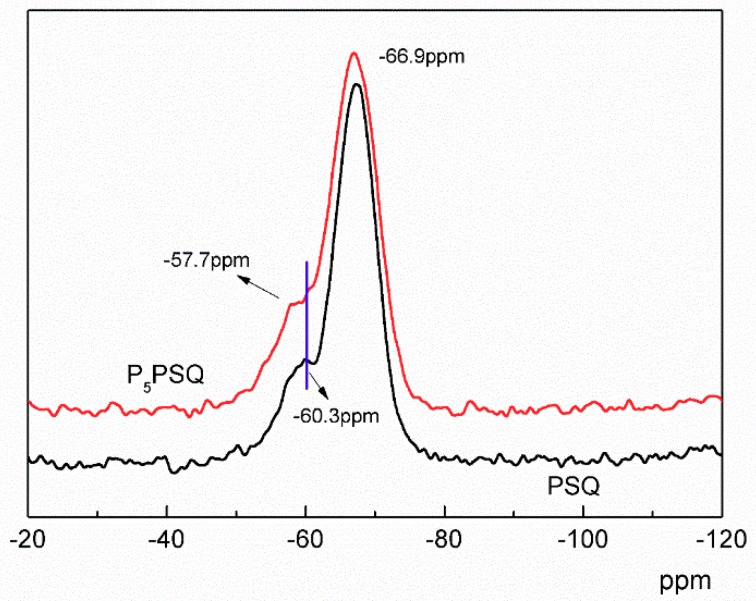
^29^SiNMR of PSQ and P_5_PSQ.

Figure 2 presents ^29^SiNMR spectra of PSQ and P_5_PSQ, providing further evidence for the reaction of phosphate with PSQ. According to the research of Han [31], the ^29^SiNMR of the poly(amino-epoxy)silsesquioxane has one main peak and a weak shoulder peak. The main peak indicates that the PSQ has a fully condensed and regular T3 structure, and the weak shoulder shows that there are amino groups. As is shown in Figure 2, the synthesized PSQ has a main peak at −66.9 ppm and a weak shoulder at −60.3 ppm, which shows that poly(amino-epoxy)-silsesquioxane has been synthesized. Furthermore, there is another shoulder in P_5_PSQ at −57.7 ppm, which indicates that the chemical environment of Si has changed due to the grafting of phosphate.

**Figure 3:**
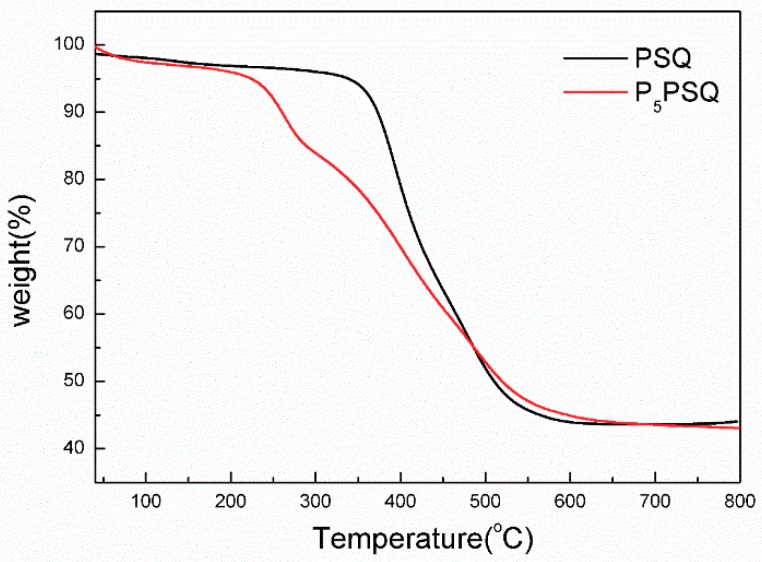
TGA curves of PSQ and P_5_PSQ under nitrogen.

Figure 3 depicts the TGA curves of PSQ and P_5_PSQ in nitrogen. Based on the results, it can be found that PSQ decomposes in only one stage and the *T*_onset_ (as measured by the temperature at which 5% of the mass has been lost) is 374 °C. P_5_PSQ has two stages in its decomposition and the *T*_onset_ decreases to 253 °C. The decrease of *T*_onset_ and the first weight loss stage are related to the presentation of organic groups like phenyl in P_5_PSQ. The second weight loss stage of P_5_PSQ is the thermal decomposition of polysilsesquioxane, which is similar to the TG curve of PSQ. The result indicates the successful synthesis of P_5_PSQ. 

### 3.2. Flame Retardant and Thermal Stability Performance of PLA

Limiting oxygen index (LOI) was used to evaluate the flammability of the PLA blends. LOI of Neat-PLA, PSQ-PLA, P_5_PSQ-PLA are shown in Table 2. The higher LOI value is, the more difficult it is for combustion to occur. Neat PLA is inflammable with a LOI value of 19.2%. With the incorporation of the flame retardant PSQ into PLA, the LOI value increases to 22.8% remarkably. The addition of P_5_PSQ further increases the LOI value to 24.1%.

**Figure 4:**
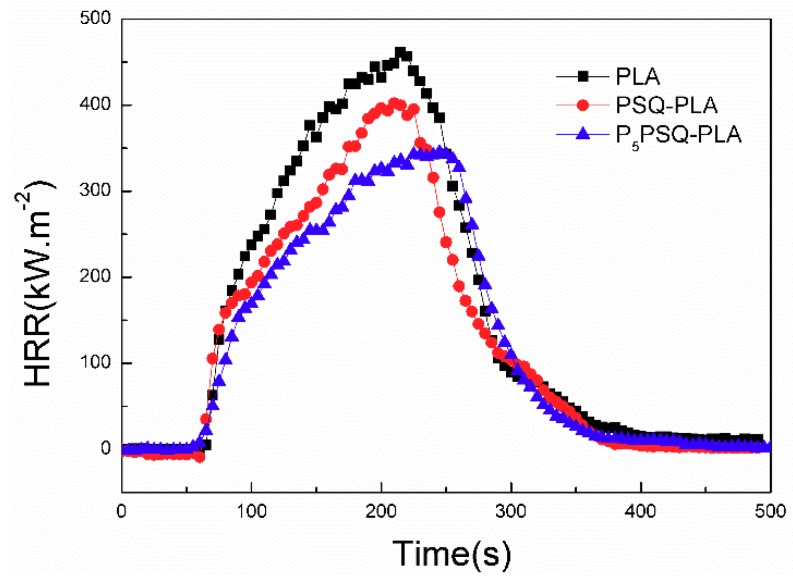
Heat release rate versus time curves of PLA and its flame retardant composites.

**Figure 5:**
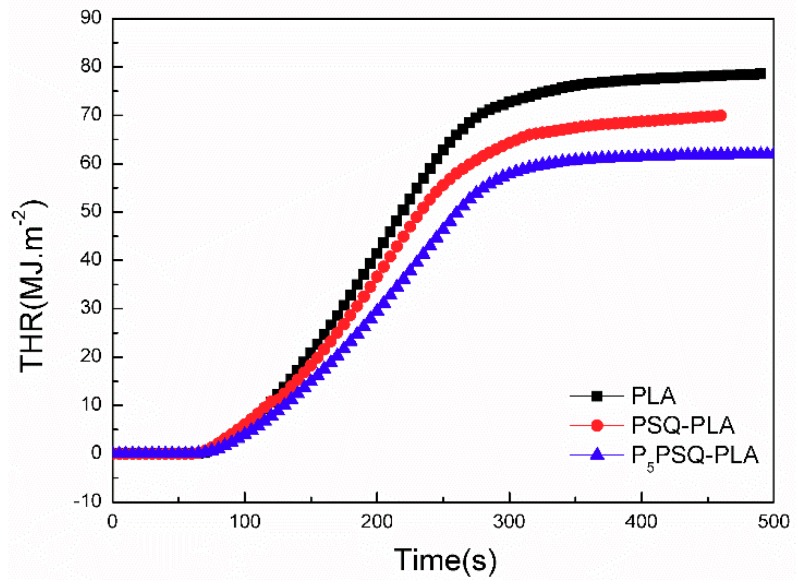
Total heat release versus time curves of PLA and its flame retardant composites.

Cone calorimetry test (CCT) is frequently employed to study the fire retardant performance of polymer materials [32], which provides information involving the heat release rate (HRR) including peak value (PHRR), time to ignition (TTI), total heat release (THR), residue mass, etc. Figure 4 and Figure 5 present the HRR vs time curves and THR vs time curves, respectively. The detailed parameters obtained in the cone tests at the heat flux of 35 kW/m^2^ are listed in Table 2. 

The data shown in Table 2 indicate that neat PLA shows limited fire resistance and displays a PHRR of 461 kW·m^−2^ and a THR of 78 MJ·m^−2^, respectively, and the residual carbon ratio is only 3.3%. The addition of PSQ induces an inconspicuous reduction in PHRR and THR, respectively. The values of PHRR and THR are decreased to 407 kW·m^−2^ and 67 MJ·m^−2^, and the decrease ratio reaches 11.7% and 14.1% compared with those values of neat PLA. Furthermore, the addition of P_5_PSQ induces a remarkable reduction in PHRR and THR. THR value decreases to 61 MJ·m^−2^, which is 21.8% lower than that of neat PLA. PHRR value decreases to 345 kW·m^−2^ with a 25.2% reduction compared to that of neat PLA. Those results show that PSQ and P_5_PSQ has effective flame retardancy in PLA, especially P_5_PSQ.

**Figure 6:**
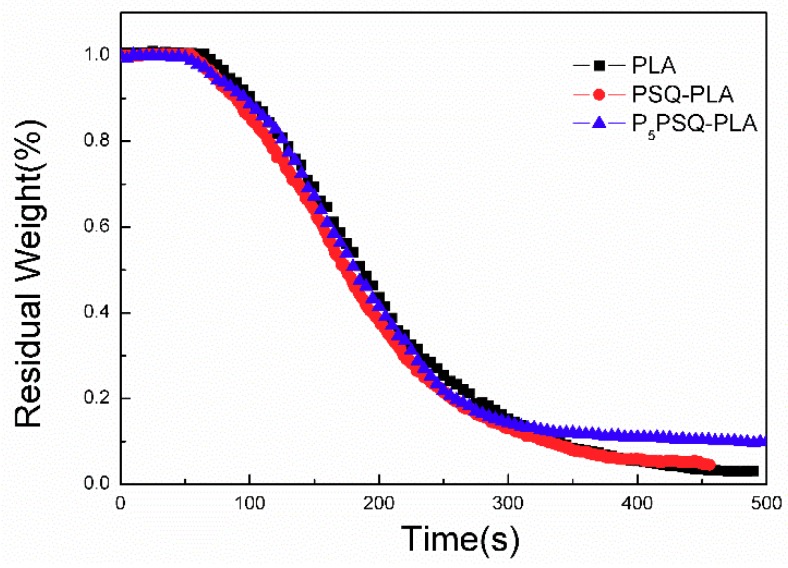
Residual mass rate versus time curves of PLA and its flame retardant composites.

Figure 6 shows the residual mass rate vs time curves in cone calorimetry test; the data is indicated in Table 2. The results show that the introduction of PSQ and P_5_PSQ induces the residual char increase to different content. The residual char ratios of PSQ-PLA and P_5_PSQ-PLA increase to 4.8% and 9.5% respectively, which are much higher than that of neat PLA. This increased carbon residue may be responsible to the promotion of PSQ and P_5_PSQ for the flame-retardancy of PLA. 

**Figure 7:**
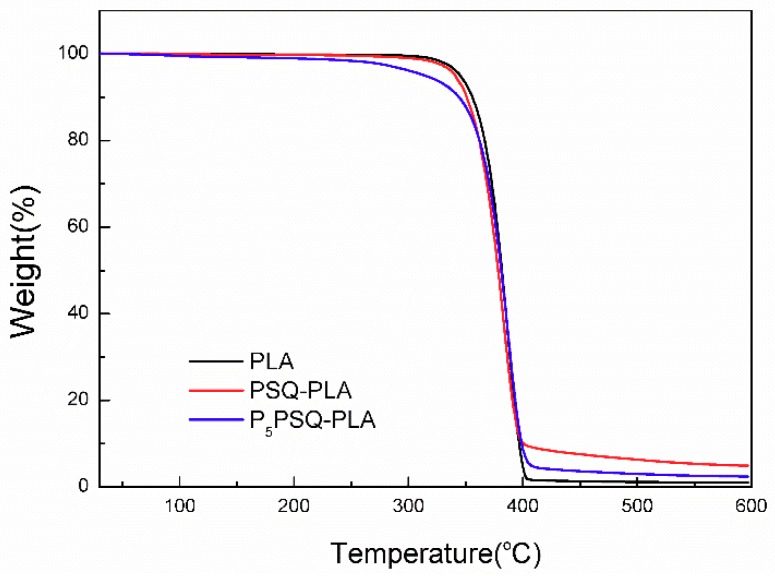
TG curves of neat PLA and its flame retardant composites.

**Figure 8:**
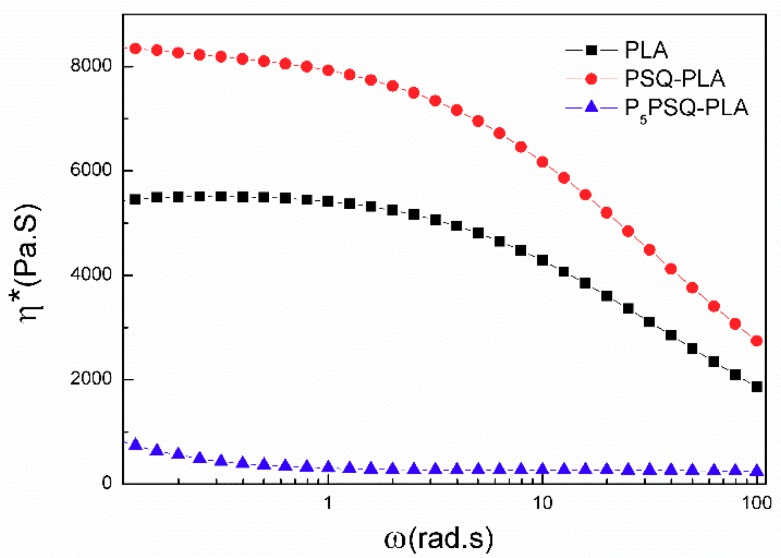
Complex viscosity of PLA and its flame retardant composites.

The typical TG curves of PLA and it’s blends in nitrogen are shown in Figure 7. As for neat PLA, the *T*_onset_ is 341 °C. And the *T*_onset_ for PSQ-PLA is 344 °C, which is a little bit higher than that of neat PLA. After the addition of the P_5_PSQ in PLA, a decreased *T*_onset_ is appearing at 323 °C, which is obviously lower than that of neat PLA. That may attribute to the improvement of P_5_PSQ to the degradation of PLA, and it is verified by the complex viscosity (η*) curves of PLA and P_5_PSQ-PLA versus frequency shown in Figure 8.

The results of Figure 8 indicate that η* values of P_5_PSQ-PLA are much lower than those values of neat PLA, while η* values of PSQ-PLA are higher. The decreased η* values of P_5_PSQ-PLA in the entire frequency indicate that there is more obvious degradation for P_5_PSQ-PLA, which is related to the improvement of phosphate for the degradation of PLA [33], so P_5_PSQ in PLA makes PLA form more carbon layer. That is responsible for the decreased TTI in cone tests as well and can also explain why P_5_PSQ-modified PLA has a denser and more regular carbon layer.

Furthermore, it is very interesting that the residue content of P_5_PSQ-PLA is lower than that of PSQ-PLA in TGA, but higher in cone calorimeter testing, which is related to the testing atmosphere and the improvement of P_5_PSQ for the carbon layer during combustion. First, as far as P_5_PSQ is concerned, the weight percent of organic groups just like phenyl group is more than that of PSQ. The groups are not stable, and they will decompose during TG analysis in nitrogen, so the residual mass of P_5_PSQ-PLA is lower than that of PSQ-PLA. Then, the cone-calorimetric test was conducted in air and it’s a combustion process. The residual mass is related to residual carbon layer. The presence of phosphorus in P_5_PSQ is beneficial to the migration of silicon element to the surface and form denser carbon layer including silicon, phosphorus and oxygen, which will be verified by the elementary analysis for carbon layer in Section 3.3. More silicon and phosphorus combine with oxygen to form carbon layer in P_5_PSQ-PLA, so the residual mass of P_5_PSQ-PLA is higher than that of PSQ-PLA in cone-calorimetric test. 

### 3.3. Morphology and Composition of Carbonaceous Foam

#### 3.3.1. Morphology of Carbon Layer

The structure of carbon residue formed on the surface of the combustion product during polymer combustion has a very important influence on the combustion behavior of the polymer. If the carbon residue structure is dense, it will isolate outside air into the combustion area and prevent the transfer of small molecules and heating into outside. On the contrary, if the residual carbon is loose, it can’t obtain flame retardancy through the good barrier effect just as the denser carbon residue, so the combustion performance of the material can’t be improved [34].

**Figure 9:**
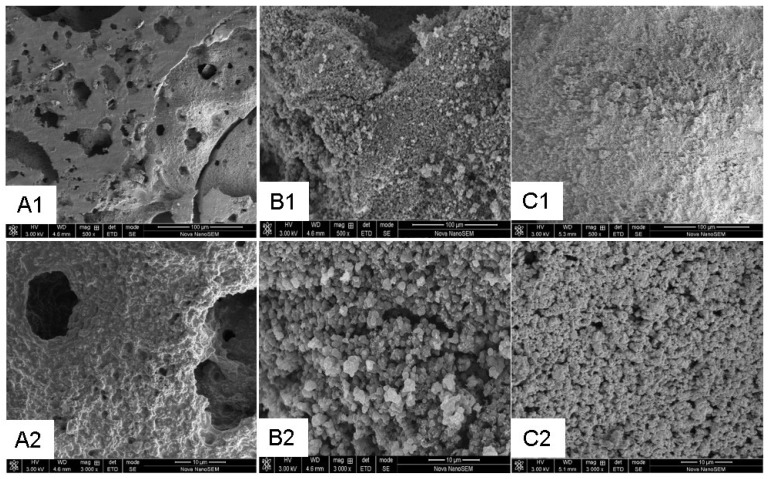
SEM images of the char residues of PLA and its flame retardant composites. 500×, 3000× (**A1**,**A2**) of the residues for PLA; 500×, 3000× (**B1**,**B2**) of the residues for PSQ-PLA; 500×, 3000 × (**C1**,**C2**) of the residues for P_5_PSQ-PLA.

The morphologies of the char residues, which were obtained from cone calorimeter test are observed by SEM. As shown in Figure 9, the char residue of neat PLA sample presents a thin and disconnected char layer. There are some voids or cracks on the surface of the carbon. The char layer does not isolate the external heat and oxygen. For PSQ-PLA and P_5_PSQ-PLA samples, the char layers become much denser and more regular than that of neat PLA sample, indicating that the introduction of PSQ or P_5_PSQ can strengthen the char layer. Comparing the char layers of PSQ-PLA and P_5_PSQ-PLA samples, the P_5_PSQ-PLA sample shows much denser char residue morphology. The denser char plays an important role in hindering the flammable gases and heat exchange by providing a good flame shield for the underlying polymer. The much denser morphology of the char residues relates to the char composition.

#### 3.3.2. Component and Structure Analysis of Residue Char

**Figure 10:**
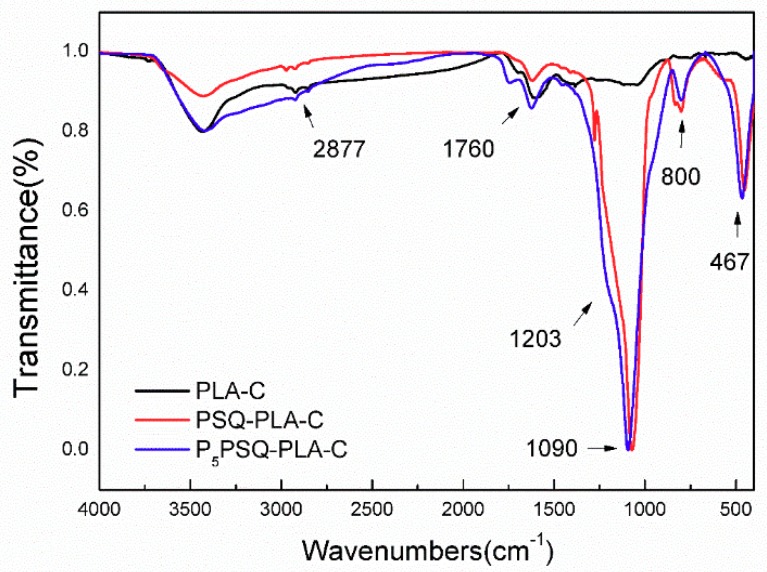
FT-IR spectra of the char residues of PLA and its flame retardant composites.

Figure 10 is the FT-IR spectra of char residue of PLA composite samples. For all the three samples, there are absorption peaks at 2877 and 1760 cm^−1^ assigned to C–H and C=O stretching vibration left by the incomplete combustion of the PLA and PLA composites. 

For PSQ-PLA and P_5_PSQ-PLA samples, some other peaks appear. First, there are new peaks at 467 and 1090 cm^−1^, which relate to the absorption of Si–O. Then, there is the absorption of Si–C at 800 cm^−1^. Furthermore, a shoulder peak around 1203 cm^−1^ is observed in the spectrum of P_5_PSQ-PLA sample, which relates to the stretching vibration of P=O. All the results show that the addition of PSQ and P_5_PSQ helps the carbon layer including Si–C structure form to improve the compactness of the carbon layer, which prevents the combustion of PLA. However, there is more char layer formation for P_5_PSQ-PLA after combustion. As a result, P_5_PSQ-PLA shows the better flame retardancy than PSQ-PLA, which may relate to the promotion of phosphorus for the char layer formation. In order to verify the deduction, the surface element content and total element content were characterized. The results are shown in Table 3.

As shown in Table 3, for P_5_PSQ-PLA, the content of phosphorus and silicon on the surface of char residue increase from the total values of 5.3% and 16.0% to 14.7% and 35.8%, respectively. The silicon content of PSQ-PLA sample on the surface of char residue increases from the total value of 19.3% to 32.7%. The results indicate that the phosphorus and silicon elements in the residual carbon obviously migrate to the surface in the combustion. The increased content ratio of silicon from total to surface is 123% in P_5_PSQ-PLA sample, which is higher than that of 69% in PSQ-PLA. Therefore, it indicates that the introduction of phosphorus is beneficial to the migration of silicon to form denser and more regular char surface. Furthermore, based on the results of FT-IR, we can know that the carbon in the surface includes Si–O–Si and Si–C, which improve the denseness of carbon layer and insulate the entrance of oxygen.

**Figure 11:**
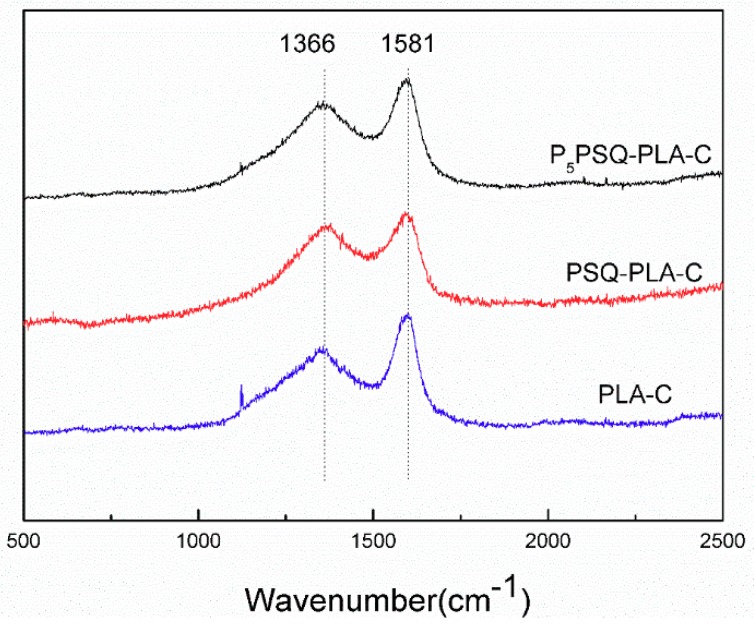
Raman spectra of the char residues of PLA and its flame retardant composites.

Moreover, in point of char layer just including carbon, the stability of char layer relates to the graphitization [35,36]. For P_5_PSQ-PLA, there are more Si–C structures, and the stability does not only relate to the graphitization. Raman spectroscopy was used to characterize the formation of graphitized carbon [37], and the results are shown in Figure 11. It is known that the peak around 1366 cm^−1^ originates from the amorphous carbon and lattice defects, and it is called as D band. The peak around 1581 cm^−1^ derives from the stretching vibration of C=C in the ordered graphitic carbon and is assigned as G band [19]. The intensity ratio R of I_G_/I_D_ symbolizes the degree of graphitic structure. By calculation, we know that the ratio values of PLA, PSQ-PLA and P_5_PSQ-PLA are 1.27, 1.21 and 1.14. The ratios of PSQ-PLA and P_5_PSQ-PLA samples are slightly lower than that of neat-PLA, which indicates the presentation of PSQ and P_5_PSQ does not make char form more graphitized structure. However, the carbon layer of PSQ-PLA and P_5_PSQ-PLA samples also contains Si–O–Si and Si–C structures. Therefore, although the graphitization of the carbon layer is not improved, the more compact and regular carbon layer has been formed due to the presentation of special Si–C and Si–O–Si structure which obtains effective shielding protection for polymer in combustion. 

### 3.4. Gas Phase Flame Retardant Mechanism

**Figure 12:**
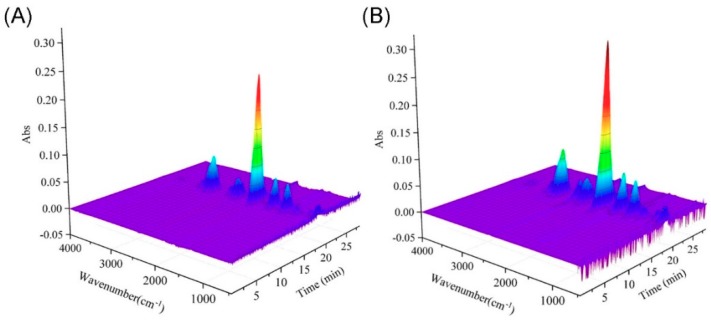
3D images of evolved gas products for (**A**) neat-PLA and (**B**) P_5_PSQ-PLA.

**Figure 13:**
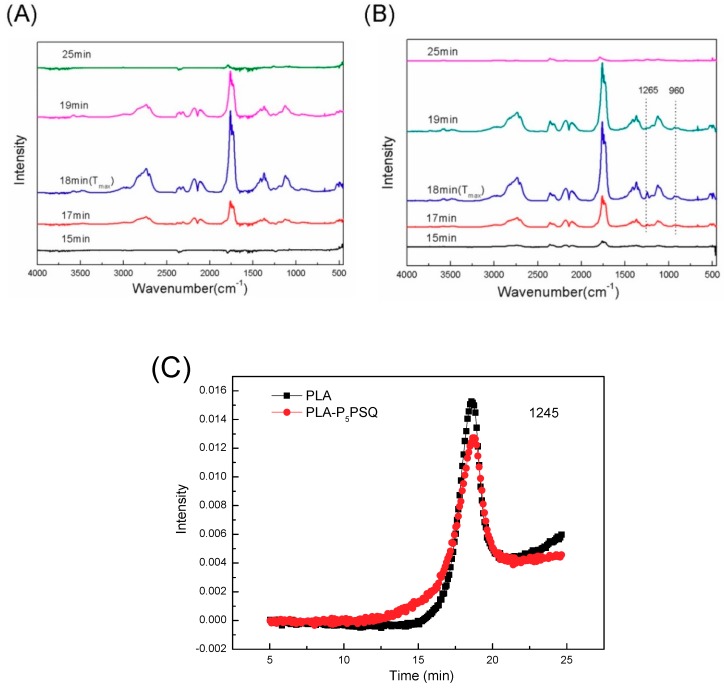
FT-IR spectra of pyrolysis products for (**A**) i-PLA, (**B**) P_5_PSQ-PLA at different times and the product curves at 1245 cm^-1^ vs time for neat-PLA and P_5_PSQ-PLA (**C**).

The pyrolysis volatile products of neat PLA and P_5_PSQ-PLA in thermal degradation were characterized by TG-IR technique to study the flame retardant action in gas phase. The 3D images of neat PLA and P_5_PSQ-PLA are shown in Figure 12, and the IR absorbance intensity of every peak at different time are shown in Figure 13.

From Figure 12 we can see that the absorptions for most of peaks enhance when P_5_PSQ is added into PLA, indicating that most pyrolysis products are released into the gas phase, especially carbonyl compounds (1760 cm^−1^), due to the improvement of phosphate in P_5_PSQ for the degradation of PLA [33]. The result is verified by the results obtained from Figure 13 further. Figure 13A shows that there are not any IR absorbencies for PLA at 15 min, indicating that there is no obvious degradation of PLA. However, as for P_5_PSQ-PLA seen in Figure 13B, some gaseous decomposition products such as carbon monoxide (2190 cm^−1^), carbon dioxide (2360 cm^−1^) and carbonyl compounds (1760 cm^−1^), are detected at the same time, which exhibits that PLA has early degradation at the presence of P_5_PSQ and coincides with the results based on Figure 12. And the earlier degradation will result in the decrease of TTI in cone tests. At 18 min (the time for the *T*_max_ happens), the gaseous products for neat PLA and P_5_PSQ-PLA are all characterized by typical strong FT-IR signals, such as water (3550 cm^−1^), hydrocarbons (2780–2950 cm^−1^), carbon monoxide, carbon dioxide, carbonyl compounds, and the infrared absorption intensities for the two samples are the strongest. It indicates there are the most obvious degradations in PLA and P_5_PSQ-PLA at this time. Furthermore, two new peaks at 960 and 1265 cm^−1^ of gaseous products in IR spectra at *T*_max_, which are caused by the P–O–C and P=O stretching vibration relatively [18,30], are observed for P_5_PSQ-PLA. This means the phosphate in P_5_PSQ emits phosphorus-containing compound. It was reported the evolution of compounds containing phosphorus could inhibit the formation for some products during the combustion of PLA [38,39], thus they may has effective flame retardancy in gas phase to a certain extent. 

Moreover, from Figure 13C, we can see that the absorption of peaks at 1245 cm^−1^ decreases when P_5_PSQ is added into PLA, which indicates that less C–O containing products are evolved into the gas phase. The results show that the inclusion of phosphorus-silicon flame retardant P_5_PSQ may restrain the release of C–O containing products [40,41], so P_5_PSQ can improve effective flame retardancy for PLA in gas phase to some extent.

## 4. Conclusion

In summary, a novel phosphorus-silicon flame retardant (P_5_PSQ) was synthesized via grafting of phosphate to polysilsesquioxane (PSQ), and it was used to increase the flame retardancy of PLA. The results indicate that phosphorus-silicon synergistic system has excellent flame-retardant properties. It can make the PHHR and THR values of PLA decrease 21.8% and 25.2% compared to the values of neat PLA in cone calorimetric test. And LOI increases from 19.2% to 24.1%. From the analysis of SEM, FT-IR, Raman, rheological analyses, element content and TG-IR analysis, a conclusion is drawn. In the condensed phase, the presence of phosphorus is beneficial to the migration of silicon element to the surface and form denser carbon layer including silicon. The carbon layer has efficient flame retardancy through the inhibition for the transfer of heat, oxygen and small molecules. In gas phase, the phosphate in P_5_PSQ emits phosphorus-containing compound that can restrain the release of C–O containing products, which can improve effective flame retardancy for PLA in gas phase to a certain extent.

## Figures and Tables

**Table 1:** Elemental compositions of PSQ and P_5_PSQ.

Samples	N (wt %)	P (wt %)	Si (wt %)
PSQ	3.5	0	14.1
P_5_PSQ	3.4	2.4	13.7

**Table 2:** LOI value and CCT data for neat PLA and its flame retardant composites.

Sample	LOI (%)	TTI (s)	PHRR (kW·m^−2^)	THR (MJ·m^−2^)	Residual Mass (wt %)
Neat-PLA	19.2	58	461	78	3.3
PSQ-PLA	22.8	54	407	67	4.8
P_5_PSQ-PLA	24.1	47	345	61	9.5

**Table 3:** Surface and total element content of char residual of P_5_PSQ-PLA.

Element	P_5_PSQ-PLA (wt %)		PSQ-PLA (wt %)	
	Surface	Total	Surface	Total
C	16.5	58.6	38.7	59.2
O	32.9	19.8	28.5	18.2
Si	35.8	16.0	32.7	19.3
P	14.7	5.3	-	-

* The “total element content” means the element content in the entire carbon layer (including inside and surface carbon). The results were obtained through elemental analysis and ICPOES analysis of the carbon layer.
